# Microorganisms in Organic Food-Issues to Be Addressed

**DOI:** 10.3390/microorganisms11061557

**Published:** 2023-06-11

**Authors:** Aparna P. Murali, Monika Trząskowska, Joanna Trafialek

**Affiliations:** Department of Food Gastronomy and Food Hygiene, Institute of Human Nutrition Sciences, Warsaw University of Life Sciences (WULS), Nowoursynowska 159C, 02-776 Warsaw, Poland; aparna_p_murali@sggw.edu.pl (A.P.M.); joanna_trafialek@sggw.edu.pl (J.T.)

**Keywords:** organic food, food safety, microorganisms, pathogen, probiotic

## Abstract

The review aimed to analyse the latest data on microorganisms present in organic food, both beneficial and unwanted. In conclusion, organic food’s microbial quality is generally similar to that of conventionally produced food. However, some studies suggest that organic food may contain fewer pathogens, such as antibiotic-resistant strains, due to the absence of antibiotic use in organic farming practices. However, there is little discussion and data regarding the importance of some methods used in organic farming and the risk of food pathogens presence. Concerning data gaps, it is necessary to plan and perform detailed studies of the microbiological safety of organic food, including foodborne viruses and parasites and factors related to this method of cultivation and specific processing requirements. Such knowledge is essential for more effective management of the safety of this food. The use of beneficial bacteria in organic food production has not yet been widely addressed in the scientific literature. This is particularly desirable due to the properties of the separately researched probiotics and the organic food matrix. The microbiological quality of organic food and its potential impact on human health is worth further research to confirm its safety and to assess the beneficial properties resulting from the addition of probiotics.

## 1. Introduction

Organic agriculture is one of the fastest-growing agricultural sectors [1] and is different from conventional agriculture in its approach to farming practices, with organic agriculture prioritizing natural methods and minimizing synthetic inputs, while conventional farming relies heavily on synthetic chemicals as well as genetically modified organisms to achieve high yields [2,3,4]. Generally, organic foods are products derived from organic agriculture, and as a consequence, these products lack Genetically Modified Organisms (GMOs), artificial colours, preservatives, and flavour enhancers. Organic cultivation avoids or prohibits the utilization of artificial feed additives, pesticides, herbicides, growth hormones, and similar substances [5,6,7,8,9]. Furthermore, one of the fastest-expanding sectors presently is the worldwide organic food market, experiencing a growth rate of several per cent annually [7,10]. This can be attributed to the overall progress of the organic farming industry and the societal focus of national policies. There is a shift towards promoting sustainable development in rural areas through the adoption of resource-conserving technologies [10].

Consumers buy organic foods because they associate this kind of food with a healthy and sustainable diet and lifestyle [10,11,12,13]. Global sales of organic products have increased significantly in the 21st century. For instance, according to a survey conducted in 2016, the majority of Americans (68%) indicated that they had bought organic food at least once during the previous month [7]. Between 2014 and 2018, organic retail market in the European Union (EU) and across Europe has seen strong progress. Every year in that period, the EU market grew by 3.4 to 3.5 billion Euros [14]. Between 2016 and 2020, the organic food market in the Asia–Pacific region experienced a compound annual growth rate (CAGR) of 11.4%, resulting in total revenues of $16.4 billion in 2020 [15].

The notion that organic food might be healthier has some support [16,17]. While there seems to be minimal variation in terms of macronutrient content (such as protein, fat, carbohydrate, and dietary fibre) between organic and conventional food products, additional differences in composition have been observed These contain higher antioxidant concentrations (particularly polyphenols) in organic crops [16,18], increased levels of omega-3 fatty acids in organic dairy products [11,16] and better fatty acid profiles in organic meat products [3,16]. When contrasting organic goods with alternatives made using conventional methods, consumers of organic food take several non-monetary factors into account [19]. Food safety, nutritional, and sensory features are these non-monetary factors that influence consumers’ preferences between organic and conventionally grown produce [19,20]. Product flavour, shelf-life, and freshness are other attributes that contribute to purchasing decisions of organic shoppers [19,21,22]. Several studies revealed that organically grown foods have lower nitrate content and higher mineral and dry matter contents than non-organically grown foods [19,23,24]. Total daily nitrates intake in vegetables from organic farming was equal to 1.91 mg NO3 kg^−1^ bw day^−1^ (28.5% ADI) [25]. In most cases, the majority of the time, the nitrate content of organic veggies was lower than that of conventional vegetables. However, production procedures, seasonal variations, and the size of those variations can all affect the nitrate level [26]. Regarding the impact on human health, a higher frequency of organic food consumption was linked with a reduced risk of cancer [27].

For centuries, different microorganisms are used in the production of food and food ingredients mainly in the processing of wine, beer, bakery, and dairy products as well as vegetables and cereals [28].

Among valuable microbes are probiotics, i.e., “live microorganisms that, when administered in adequate amounts, confer a health benefit on the host”. Probiotics can have different means of administration, for example as drugs, with common and medicinal foods, non-oral probiotics, animal feed, defined microbial consortia, dietary supplements, or infant formula [29]. Probiotics have various health benefits, including intestinal and non-intestinal effects. Among others, the prevention of diarrhoea and gastrointestinal cancers, the alleviation of lactose intolerance, and a decrease in *Helicobacter pylori* infections. The impact of using probiotics on immunological conditions, including asthma, and atopic disorders is unconfirmed in humans. However, they may have an advantageous effect by reducing symptom severity and medication usage [30,31,32].

The other group of microorganisms present in food are spoilage and pathogenic microorganisms, which are considered as one of the main causes of food loss [33]. Moreover, foodborne illnesses due to the consumption of foods contaminated with foodborne pathogens pose a significant public health problem [34]. Like conventional food, organic food must by law fulfil the food safety requirements, and it must be produced according to specific regulations [35,36,37,38]. The microbial quality of organic food can vary depending on a variety of factors, including production practices, handling, processing, and storage conditions [39].

In 2017, Garcia and Teixeira conducted an in-depth literature review and discussion of the safety of organic food [35]. Regarding pathogens, food samples contained harmful microorganisms more often than those from conventional systems. The authors suggested further food safety studies comparing organic and conventional production with more samples analysed and over a longer period.

Therefore, the purpose of the review was to analyse the latest data on microorganisms present in organic food, not only undesirable but also beneficial. Comprehensive knowledge of the microbiological quality of organic food is essential to fully understand the factors that shape its unique value. Determining data gaps allows for proper planning of further research and, consequently, ensuring the highest microbiological quality and safety of organic food.

## 2. Materials and Methods

A comprehensive search of relevant reports from academic databases, such as PubMed, Scopus, and Science Direct, was conducted. The search was related to the keywords, such as organic food, organic food standards, microorganisms, microbial quality, food safety, pathogens, probiotics, etc.

The timeline for the literature sources was set from 2017 to 2023. The article titles and abstracts were reviewed and duplicates were eliminated. Only studies on organic food quality and safety and microorganisms were considered for inclusion. Selected sources of evidence included research and review articles, short communications, peer-reviewed conference materials, book chapters, and to a minimum extent, websites of recognized institutions.

## 3. Results

### 3.1. Standards for Organic Food

Some standards are referring to the set of guidelines and regulations that define what qualifies as organic food. Furthermore, they ensure that organic food is produced in a way that minimizes harm to the environment and promotes sustainable agriculture. In this context, various regulatory bodies around the world have developed their organic food standards, which are designed to meet the unique needs of their respective countries and regions. Whenever doubts exist relating to the usage of synthetic chemicals, the relevant law should be consulted.

The USDA (United States Department of Agriculture) [36,37,40,41] and the EU (European Union) [38,42] have established stringent standards for organic foods, ensuring the integrity and quality of these products. In Asia, the standards for organic foods vary among countries. While some Asian countries have developed their organic certification systems, there is no unified standard across the region. A regional standard for organic production in East, Southeast, and South Asia is called the Asia Regional Organic Standard (AROS). The procedure that led to AROS was intended to harmonize regional organic standards and promote new ones [43,44].

Legal requirements do not define any additional precautions for food products regarding microbiological quality. However, care must be taken with plant and animal components to preserve or improve soil organic matter in a way that avoids the transmission of pathogenic organisms into crops, soil, or water [45].

Microbial quality is an essential parameter that determines the safety and shelf-life of food products [45,46]. Organic food should not be treated with chemical preservatives, which can increase the risk of bacterial growth during storage and transportation. However, there are allowed natural substances and processes that are used along the organic food chain to ensure food safety. Moreover, consumers need to handle and prepare organic food safely to minimize the risk of microbial contamination [47]. The presence of harmful microorganisms, such as pathogenic bacteria, viruses, and parasites, in food can cause foodborne illnesses, leading to severe health consequences [48,49,50]. Therefore, ensuring the microbial safety of organic food is very important.

In the EU, from a legal point of view, Commission Regulation (EC) No. 2073/2005 of 15 November 2005 and Commission Regulation (EC) No. 1441/2007 of 5 December 2007 on microbiological criteria for food products lay down microbiological criteria for certain microorganisms and the implementing regulations to be met by food stakeholders. The regulation sets limits on the levels of microorganisms that are considered acceptable in organic food products based on the potential health risks associated with their consumption. These limits are based on scientific evidence and are designed to ensure that organic food products are safe for consumers to eat. In addition to setting limits on microbial contamination, the regulation also requires organic food producers to implement good hygiene practices and monitor and test their products for microbial contamination. This helps to further ensure that organic food products meet the microbial quality standards established by the EU [51,52].

### 3.2. Microorganisms in Organic Food

Microbial contamination in organic food production can pose health risks to consumers as it may lead to foodborne illnesses, such as bacterial infections, viral outbreaks, and fungal infections. Vulnerable populations, such as pregnant women, elderly individuals, and those with weakened immune systems, may be particularly susceptible to these risks [53].

Microorganisms can be present in any type of food, including organic food. Microbes are found all over the globe with a few exceptions, for example, sterilized surfaces [54]. Microorganisms such as *Salmonella* spp., *Escherichia coli* O157:H7, and *Listeria* spp. are commonly associated with plant foods. *Salmonella* spp. is frequently found in cut fruits, pre-cut melons, fresh papayas, cucumbers, and other fresh produce [55,56,57]. *Escherichia coli* O157:H7 is frequently associated with sprouts, lettuce, spinach, and other leafy greens [56,58,59,60]. *Listeria* spp. are often found in cantaloupes, mushrooms, apples, stone fruits, and other fresh produce [56,61,62,63]. *Salmonella* Typhimurium is a type of bacteria commonly found in animal foods such as poultry, beef, pork, and seafood [64,65,66]. *Campylobacter jejuni* and *Campylobacter coli* are two species of bacteria widely found in animal foods such as poultry, cattle, pigs, and even domestic pets [64,67]. Shiga-toxin-producing *E. coli* (STEC) is another type of bacteria that can be found in animal foods such as beef, sheep, goats, and other ruminants [64,68]. *Listeria monocytogenes* is a microorganism that can be found in animal foods of cattle, sheep, goats, and poultry origin [64,69,70]. These microorganisms can cause foodborne illnesses and pose a risk to public health. To reduce the risk of foodborne illness, it is crucial to follow proper food safety practices when growing, processing, and preparing food.

Beneficial microorganisms that benefit human health and food production consist of another kind of microorganisms found in food. Numerous clinical studies have demonstrated the efficacy of probiotics in the treatment of conditions, such as type 2 diabetes, obesity, non-alcoholic fatty liver disease, and insulin resistance syndrome. Probiotics’ beneficial benefits on human health have also been demonstrated by raising immunity (immunomodulation) [71]. For example, *Bifidobacterium breve* is commonly found in European fermented milk [72,73]. *Enterococcus faecium* is present in soybean, dairy, meat, and vegetables, and is used in probiotic foods to support gut health. *Lactobacillus acidophilus* is found in fermented milk and vegetables. Moreover, *Lactobacillus delbrueckii* is commonly found in yoghurt, fermented milk, and mozzarella cheese, and can improve digestion and promote overall health [72]. *Lactobacillus kefiri* is found in fermented milk and can reduce the bitter taste in citrus juice [72,74,75]. *Pediococcus acidilactici* is used in meat fermentation and biopreservation of meat as well as in cheese starters [72,76]. *Lactococcus lactis* is used as a dairy starter and can produce nisin that plays a role of a protective culture that helps to prevent the growth of harmful bacteria in food [72].

Given the high interest in organic food, little data on its microbiological quality has been found in the available literature as of 2017. The newest and most available data are listed in Table 1.

The study of Urkek et al. (2017) aimed to investigate the effects of production methods and milk collection periods on the somatic cell count and some microbiological properties in Turkey. Comparing organic milk to conventional milk, the general means of the total aerobic mesophilic bacteria, coliform, yeast, and mould counts were significantly lower, but the general means of the somatic cell count and the coagulase-positive *S. aureus* count was significantly higher [77].

Malissiova et al. (2017) studied the differences in the microbial profile and antimicrobial resistance of bacteria isolated from milk from organic and conventional sheep and goat farms. The study involved 25 organic and 25 conventional sheep and goat farms. It was found that milk from organic farms has a better microbiological profile compared to milk from conventional farms [78].

Another research regarding the dairy product was conducted by Selah et al. (2023). The goal of this study was to assess the microbiological quality of organic and conventional Minas Frescal cheese samples as well as the antimicrobial susceptibility of isolated cultures of *Escherichia coli* and coagulase-positive staphylococci to various antimicrobial agents. *Listeria* spp. and *Salmonella* spp. were not detected in any of the analysed samples. For coagulase-positive staphylococci, 43.4% conventional and 26.6% organic samples had a higher count than recommended by Brazilian regulations but with no significant difference between the systems. In determining the most likely *E. coli* count, a significant difference was observed between the systems, with a higher rate of contamination in cheeses from the organic system. Given the similarity of the acquired results, it is required to investigate other criteria, such as the production system, herd health, and appropriate production methods, to thoroughly compare the two systems [79].

Regarding plant organic foods, Szczech et al. [80] have found that the numbers of mesophilic bacteria, yeasts and moulds, coliforms, and Enterobacteriaceae for radishes and carrots were similar in organic and conventional farming systems. Organic lettuce contained significantly more bacteria than conventional lettuce. Organic beetroot contained higher amounts of yeasts and moulds and Enterobacteriaceae than conventional. Vegetables from organic farms were characterized by a significantly higher load of *E. coli* than vegetables from conventional farms. Furthermore, a farmer survey was conducted to gather information on farm management practices. An index (from 0—no risk to 4—high risk) of potential contamination of the product with human pathogens was developed related to the fertilization system. Its value increased with the increased share of manure and other animal waste used for fertilization. Organic products, therefore, had a higher contamination risk index (2–4) than conventional vegetables (1–2). High rates were associated with a higher amount of E. *coli*. It was discovered that the organic farm’s fertilizer strategy may degrade the hygienic quality of the products. [80].

Kuan et al. [81] compared the microbiological status of organic vs. conventional fresh produce at the retail level in Malaysia. The findings revealed that mesophilic aerobic bacteria, yeasts and moulds, and all coliforms were present in comparable amounts in most types of conventional and organic vegetables. None of the samples examined for this investigation included *E. coli* O157:H7 or *S.* Typhimurium. Vegetables cultivated either organically or conventionally did not generally provide a higher microbial risk. [81].

In terms of research on the presence or development of organic foods with the addition of beneficial microorganisms (defined as probiotics), no studies were found. The only example in this field is the study by Rzepkowska et al. [82] on the isolation of strains with potential probiotic properties. Thus, spontaneously fermented organic foods can be a source of beneficial microorganisms. This direction of research is supported by the results of Wassermann et al. [83] who compared the apple microbiota to find tissue-specific variations and the effects of organic vs. conventional management. Organic and conventional apples contained a similar quantity of microbiota. However, organically managed apples harbour a significantly more diverse, more even, and distinct microbiota compared to conventional ones.

In light of numerous studies on the positive impact of microorganisms on health, including gut microbiota, it is important to address this issue in organic food research. Many factors influence the composition of the gut microbiota and its functioning, but one of the main triggers is diet [84]. Therefore, one can expect additional benefits that may result from combining in one product the proven properties of organic food, e.g., antioxidants, dietary fibre content, and others [16,18,19], with the proven impact of food ingredients on the condition of the microbiota [85,86,87].

**Table 1:** Examples of pathogens and beneficial organisms found in organic foods since 2017.

Pathogen	Animal Foods	Count [log10 cfu g/mL^−1^] or Prevalence [%]	Reference
*Campylobacter*	Raw chicken meat	25%	[88]
*Salmonella*	Pigs Eggs Dairy farms Poultry farms	8.3% 2.6% 20% 2.9%	[88,89]
Shiga toxin-producing *Escherichia coli*	Beef	14.8%	[88,89]
*Listeria monocytogenes*	Chicken meat Eggs	25% 1.8%	[88]
*Staphylococcus aureus*	Chicken farms Meat Poultry farms Dairy products Milk	1.3% 16.7% 27.4% 31% 0.28–0.91	[79,88] [77]
Coliforms	Milk	2.72–4,46	[77]
Yeasts and moulds	Milk	2.46–3.70	[77]
Pathogen	Plant Foods		
Mesophilic bacteria	Organic vegetables	6.6–7.2	[80,81]
*Enterobacteriaceae*	Lettuce High-protein bar	2.5 0 2.81–3.32	[80,81]
Coliforms	Lettuce	1.80	[80,81]
*Escherichia coli*	Organic vegetables	1.00	[80,81]
Yeasts and moulds	Organic vegetables	5.10	[80,81]
Beneficial Bacteria	Food Product		
*Lactic acid bacteria with high potential for food application*	Organic Whey	-	[82]

### 3.3. Factors Affecting the Microbial Quality of Organic Food

The main sources of microbial contamination and strategies for improving the microbial quality of organic food are summarized in Table 2.

The microbial safety of fresh produce is influenced significantly by various factors, with the conditions present at the growing site being particularly crucial. Both organic and conventional agriculture make substantial use of manure and other animal wastes. Concerns regarding the possibility of products being contaminated with microbiological organisms, particularly *Escherichia coli* O157, occur when manure is used as fertilizer in either conventional or organic agriculture [90]. Soil fertilized by animal manure is more likely to be contaminated with enteric pathogens because of their ability to survive in soil for months or years [91]. The content of *E. coli* and *Salmonella* spp. is between 10^2^ and 10^5^ CFU/g and between 10^2^ and 10^7^ CFU/g, respectively, in animal faeces. The manure of ruminants (cattle and sheep) and sewage are reputed to be the main sources of *Salmonella* spp. and *E. coli* O157:H7. Furthermore, *C. jejuni* is a typical member of the gastrointestinal microbiota of poultry, pigs, and cattle. Fresh produce’s microbiological safety is influenced by the quality of the irrigation water and the system type. The safety of fresh produce’s microbiology is influenced by harvesting and processing. These processes that can both contaminate products with hazardous microorganisms and encourage bacterial development include human and mechanical touch, immersion in water, and cutting or slicing [92].

Concerning manure usage in organic farming, the research conducted by Nazareth et al. [89] is quite interesting and relevant. The researchers examined models of integrated organic crop and livestock systems established in three US states. Organically reared cattle and their feed, faeces, skin, and meat were tested for two pathogens, *Escherichia coli* O157:H7 and *Salmonella* spp. The results confirmed that *E. coli* O157:H7 was not isolated from any skin or meat sample. Across all locations, the prevalence of *E. coli* O157:H7 in feed and faeces was 9.43 and 7.26%, respectively. *Salmonella* spp. were isolated from 1.89, 3.33, and 18.6% of feed, faeces, and skin samples, respectively. *Salmonella* spp. was not detected in any meat sample. From June to August, the feed was more likely to test positive for *E. coli* O157:H7, highlighting the necessity of stringent sanitation procedures to avoid feed contamination. These findings demonstrate the potential for reducing the risk of microbiological contamination in integrated agricultural and livestock systems through rigorous adherence to food safety management measures [89].

The effectiveness of composting chicken manure was demonstrated in the experiment of Begum et al. [93] The study investigated the current situation of small poultry farms and their waste management practices in selected areas of Bangladesh, and the presence of *Escherichia coli* and *Salmonella* in vegetables from farms using unprocessed poultry manure as fertilizer. *E. coli* or *Salmonella* or both have been confirmed in vegetables, soil and, pond water but only in untreated poultry waste used for fertilization.

### 3.4. Strategies for Improving Microbial Quality of Organic Food and Data Gaps

One of the essential components for sustaining life on Earth is soil. Almost half of the Sustainable Development Goals (SDGs) are linked to soil [94]. Numerous goals can be achieved by adopting sustainable land utilization and enhancing the quality of the soil. These objectives can be defined as the ability of soil to serve as an essential living system, supporting biological productivity, safeguarding environmental integrity, and preserving the well-being of plants, animals, and humans [94,95]. Pathogenic microorganisms originate from the environment in which plants grow as well as post-harvest movements, processing, and transport movements. One of the most important post-harvest practices for preventing food-borne illnesses and preserving the excellent quality of raw fruits and vegetables is post-harvest microbial control, for instance, utilizing suitable disinfection procedures [96].

Organic food processors need to implement rigorous food safety practices, including regular monitoring, testing, pest control, and training of personnel, to prevent or minimize microbial contamination during processing [97].

A range of food safety issues have been raised concerning organic primary production and processing, including mycotoxins, enteric pathogens, and heavy metal and agrochemical residues [98,99]. In the regulation on microbiological criteria of food products (No. 2073/2005), it is stated that food business operators at each step of food production, processing, and distribution, including retail, must implement precautions to assure that food safety and process hygiene criteria are met as part of their procedures based on Hazard Analysis and Critical Control Point (HACCP) principles along with the implementation of good hygiene practices (GHP). Therefore, the HACCP plans for organic production are elaborated and brochures for consumers, retailers, and producers on the safety and contamination of organic food are written [97]. Documentation for apples, grapes, cereals, cabbage, and tomatoes as well as milk and egg production are included. These provide practical advice to improve the quality and safety of organic products [100].

Microbial contamination can be a major concern when it comes to the packaging of organic food. The packaging stage of the food production process is essential because it maintains the quality of the finished goods for use in storage, transit, and consumption. [101]. With packaging methods, the industry has designed modified atmosphere packaging (MAP), edible-film coating, and active packaging. Packaging methods need minimal human intervention over the properties of the food product, giving consumers a sense of an “unprocessed” and “natural” product [102,103,104].

The consumer of processed organic food expects a specific nutritional and sensory value. Particularly, processing techniques that do not significantly alter the nutritional and sensory qualities of food could provide goods that look appealing and fresh. Given that they are required to maintain the nutritional value of food, health-conscious consumers should prefer “minimally” processed food [102,105,106]. It is proved that even with restricted usage of chemical substances, simple combinations of water heated to 60 °C, acetic acid (0.2–5%), and H_2_O_2_ (3–4%) can reduce the count of all three species of bacterial pathogens on mung bean [107]. Therefore, a re-examination of known methods using permitted chemicals seems to be the right direction in the development of research on the safety of organic food.

To mitigate microbial contamination during wholesale storage of organic food, proper handling, storage, and transportation practices should be followed. This includes maintaining appropriate temperature and humidity levels, regular cleaning and sanitizing of equipment and facilities, avoiding cross-contamination between different food products, and implementing strict hygiene protocols for food handlers [108,109].

Comparing organic products to conventional foods on the same level, organic products appear to be more sensitive to microbial infection [110,111]. Therefore, additional research is required to assess the product’s safety and determine the appropriate storage times [110]. Proper storage of organic food can help prevent the growth of harmful bacteria. This can include refrigeration, proper packaging, and controlling the temperature and humidity. Food products’ moisture content can fluctuate even slightly due to changes in atmospheric humidity. The increase in air humidity caused the product under test to absorb moisture from air and also caused a change in the product’s mass. At 80% relative air humidity and 25 °C, the maximum mass gain and mass growth rate were noted [112]. All strategies for improving the microbial quality of organic food should be based on the requirements of the hygiene of foodstuffs and should contain rules and programs referring to Good Manufacturing Practices, Good Hygiene Practices, and Hazard Analysis and Critical Control Point (HACCP) principles [113]. They are mandatory both for conventional and organic food producers and processors alike. The examples of the strategies for improving and managing the microbial quality of organic food at different stages are presented in Table 2.

**Table 2:** Microbial contamination sources and main strategies for improving microbial quality of organic food production.

Production Stage/Sources	Strategies for Improving Microbial Quality	Reference
Farming		
Soil, faeces, irrigation water, reconstituted fungicides and insecticides, dust, insects, inadequately composted manure, wild or domestic animals, and human handling.	Good agricultural and handling practices relating to soil, faeces, manure, pests, and cleaning. Training of workers. Post-harvest microbial control.	[53,92,108,114,115]
Harvesting		
Harvesting equipment, transport containers, insects, dust, and human handling.	Establishing rules of conduct for harvesting. Training of workers and using good agricultural and handling practices. Post-harvest microbial control.	[53,92,108,116]
Processing		
Rinse water, ice, transport vehicles, and human handling. Improper temperature control, cross-contamination with contaminated surfaces or equipment, and lack of proper packaging or sealing.	Implementing effective washing and disinfection program refers to transporting vehicles, equipment, and production utensils to avoid cross-contamination with contaminated surfaces or equipment. Pest control. Applying proper personnel hygiene rules and training of personnel. Establishment of the right conditions for storage of raw materials and final product, daily temperature control, and processing methods.	[108,113,117,118]
Wholesale storage		
Storage temperature, containers, dust, insects, human handling, storage duration etc.	Establishment of proper handling, storage, and transportation practices to maintain appropriate temperature and humidity levels. Regular cleaning and sanitizing of equipment and facilities to avoid cross-contamination between different food products, and implementing strict hygiene protocols for food handlers.	[108,113,117,118]

## 4. Conclusions

In conclusion, the microbial quality of organic food is generally considered to be similar to that of conventionally produced food. However, some studies suggest that organic food may contain lower numbers of harmful bacteria, such as antibiotic-resistant strains, due to the absence of antibiotic use in organic farming practices. Therefore, it is important to note that while organic farming practices may reduce the risk of certain bacterial infections; however, they do not eliminate all potential risks associated with foodborne illness.

On the other hand, there is still little discussion and data regarding the importance of some methods used in organic farming and the risk of food pathogens presence. From the scientific point of view, additional studies are needed to fully assess the risk of using manure and free-range breeding and its influence on the transmission of foodborne pathogens along the food chain.

The application of proper food safety measures, such as appropriate cooking and handling techniques, should always be followed regardless of whether the food is organic or conventionally grown.

Concerning data gaps, it is necessary to plan and perform detailed studies of the microbiological safety of organic food, including factors related to this method of cultivation and specific food processing requirements. Among the missing data are those on the prevalence of foodborne viruses and parasites. Such knowledge is essential for more effective management of the safety of this food.

Bearing in mind that not all microorganisms are harmful, the use of beneficial ones in organic food production has not yet been widely addressed in the scientific literature. This is particularly desirable due to the properties of the separately researched probiotics and the organic food matrix. The expected mutually reinforcing effect must be supported by research results.

In conclusion, the microbiological quality of organic food and its potential impact on human health is worth further research. Both to confirm its safety and to assess the beneficial properties resulting from the addition of probiotics.

## Data Availability

No new data were created or analysed in this study. Data sharing is not applicable to this article.
